# The Effect of Unsaturated Fatty Acids on Molecular Markers of Cholesterol Homeostasis in THP-1 Macrophages

**DOI:** 10.5812/ircmj.11780

**Published:** 2013-07-05

**Authors:** Javad Zavar Reza, Hossein Nahangi, Reza Mansouri, Ali Dehghani, Majid Mojarrad, Mohammad Fathi, Abdolrahim Nikzamir, Mir Saeed Yekaninejad

**Affiliations:** 1 Department of Biochemistry, Faculty of Medicine, Shahid Sadoughi University of Medical Sciences, Yazd, IR Iran; 2 Department of Anatomy, Faculty of Medicine, Shahid Sadoughi University of Medical, Yazd, IR Iran; 3 Department of Immunology, Faculty of Medicine, Shahid Sadoughi University of Medical Sciences, Yazd, IR Iran; 4 Department of Biostatistics and Epidemiology, Faculty of Health, Shahid Sadoughi University of Medical Sciences, Yazd, IR Iran; 5 Department of Medical Genetics, School of Medicine, Mashhad University of Medical Sciences, Mashhad, IR Iran; 6 Department of Anesthesiology, Faculty of Medicine, Mofid Children’s Hospital, Shahid Beheshti University of Medical Sciences, Tehran, IR Iran; 7 Endocrine Research Center, Valiasr Hospital, Tehran University of Medical Sciences, Tehran, IR Iran; 8 Department of Epidemiology and Biostatistics, School of Public Health, Tehran University of Medical Sciences, Tehran, IR Iran

**Keywords:** Atherosclerosis, Macrophages, Receptors, Oxidized LDL, Peroxisome Proliferator-Activated Receptors

## Abstract

**Background:**

Macrophages derived foam cells are key factors in the maladaptive immune and inflammatory response.

**Objectives:**

The study of the cholesterol homeostasis and the molecular factor involved in these cells is very important in understanding the process of atherosclerosis and the mechanisms that prevent its occurrence.

**Materials and Methods:**

This experimental study investigated the effects of c9, t11-Conjugated Linoleic Acid (c9, t11-CLA). Alpha Linolenic Acid (LA), and Eicosapentaenoic Acid (EPA) on the PPARα and ACAT1 mRNA expression by Real time PCR and cholesterol homeostasis in THP-1 macrophages derived foam cells.

**Results:**

Incubation of CLA, LA, EPA, and synthetic ligands did not prevent increasing the cellular total cholesterol (TC). Free cholesterol (FC) is increased by Sandoz58-035 (P = 0.024) and decreased by fatty acids and Wy14643 (Pirinixic acid) (P = 0.035). The pattern of distribution of %EC is similar to the EC pattern distribution. The ACAT1 mRNA expression was significantly increased by EPA (P = 0.009), but c9, t11- CLA, LA, Wy14643, and Sandoz58-035 had no significant effect on the mRNA level of ACAT1 expression compared to DMSO(Dimethyl sulfoxide).

**Discussions:**

In comparison to the control of Wy14643, Sandoz58-035, c9 and t11-CLA, EPA increased the PPARα mRNA levels (P = 0.024, P = 0.041, P = 0.043, and P = 0.004, respectively), even though, LA had no significant effect on the PPARα mRNA expression (P = 0.489).

**Conclusions:**

Variations in the chemical structure of fatty acids can affect their physiological function.

## 1. Background

Atherosclerosis is one of the main causes of death in advanced countries (1). Atherosclerosis initiates with the accumulation of apolipoprotein B-containing lipoproteins in the subendothelial of the artery (2). The blood flow carries these Lipoproteins to the intima layer and subsequently with their oxidative modification; an early inflammatory response is raised. The activation of endothelial cells leads to the recruitment of blood-borne monocytes and immigration of them to the subendothelial layer (3, 4). By the macrophage colony-stimulating factor (M-CSF) and other differentiation factors, the monocytes were differentiated to macrophage in early atherosclerotic lesions (5, 6). In the beginning of formation and progress of atherogenesis, many macrophages have many lipid droplets in the cytoplasm. The formation of these lipid-loaded macrophages or foam cells begins when they uptake and process modified Low Density Lipoproteins (m-LDL), especially oxidized LDL (ox-LDL) via scavenger receptors (7). After digestion, the cholesterol esters (CE) of the LDLs are hydrolyzed to free cholesterol and fatty acids (8). The free cholesterol can leave the cell or undergo re-esterification to CE by the ER enzyme acyl-CoA: cholesterol ester transfers (ACAT) (9). Therefore, the accumulation of cholesterol ester droplets in cells like as MQs occurs. Some studies have shown that the upregulation of the ACAT1 expression in monocytes and MQs is strongly associated with the initiation and progression of the atherosclerosis in cell culture and in experimental animal models (9-12). Fatty acids are important ligands for LXR (liver X receptor) (13) and PPAR (14, 15).These transcriptions are as a substrate for ACAT1, so different fatty acids can have different effects on these factors and influence cholesterol homeostasis in macrophage (16). The ACAT1 mRNA levels increased in macrophages compared to monocytes of the mouse liver in response to a high fat, and high cholesterol diet (17-19). Also some studies have shown that free fatty acids can regulate the ACAT1 gene expression (14, 17, 18).

## 2. Objectives

This experimental study was designed to elucidate the ability of w-fatty acids and conjugated fatty acids in the prevention of foam cell formation through the effect on the gene expression of PPARα and ACAT1.In this study, The ACAT1 mRNA expression was significantly increased by EPA, but c9, t11- CLA, LA, Wy14643, and Sandoz58-035 had no significant effect on the mRNA level of ACAT1 expression compared to DMSO.

3. Materials and Methods

### 3.1. Materials

The THP-1 cell was obtained from the IR Iranian branch of Institute Pasture. Cell culture media, Glutamine, penicillin, and streptomycin media supplement, the Serum Free Medium (SFM) and the fetal bovine serum (FBS) were obtained from the Invitrogen Corporation, Paisley, UK. Phorbol 12-myristate13-acetate (PMA), dimethyl sulfoxide (DMSO), Wy 14643, Sandoz 58-035, the linolenic acid, the eicosapentaenoic acid, and the conjugated linoleic acid were obtained from Sigma-Aldrich, The USA. Ac-LDL was purchased from calbiochem com, The USA. Reagents, kits for RNA extraction, reverse transcription and SYBER Green, and PCR Master Mix Reagents were obtained from Qiagen, The USA. All other chemicals were obtained from Sigma Aldrich, The USA. Human monocytic THP-1 cells were cultured in the RPMI 1640 medium, supplemented with a 10% fetal bovine serum (FBS), streptomycin, amphotericin B, sodium pyruvate, 2 mM L glutamine, 50 μM of 2-mercaptoethanol in a humid atmosphere containing 5% CO2 at 37°C. For experimental purposes, cells were cultured at a 1 × 106 density in a serum free media (SFM). All tests were performed in triplicate. Two expert researchers performed the tests. The inter-rater reliability between two evaluators was 0.91.

### 3.2. Cell Differentiation and Fatty Acid Treatment

For differentiation of THP-1 to macrophage, THP-1 cells were washed with serum free RPMI 1640 and resuspended in SFM in the presence of 100 ng/ml phorbol 12-myristate 13-acetate (PMA) for 72 h (19). All fatty acids and synthetic ligands were dissolved in DMSO (the final concentration was ≤ 0.1%). Cells were pretreated with fatty acids and chemical ligands before cholesterol loading for 24 h, and then for the induction of foam cell transformation, cells were incubated with a 50 μg/ml Ac-LDL in the SFM medium for 48 h (20). The concentration of fatty acids and chemical ligands in this experiment were: 100 μM for LA, EPA and CLA, 50 μM, 5 μM for Wy14643Sandoz 58- 035, respectively.

3.3. RNA Extraction and Real Time Quantitative RT-PCR Analysis

The mRNA levels were quantitated by a real-time PCR. Instruments were calibrated before conducting the trial and the inter rater reliability of two evaluators were calculated by ICC (Intra class correlation). The ICC of inter rater reliability for PCR and ELISA were 0.89 and 0.92 respectively. The total RNA was extracted from THP- 1 foam cells using the RNA kit (Qiagen, The USA). RNA was stored at -70°C until real-time polymerase chain reaction (PCR) occurred. RNA for real time PCR, was reversed transcribed according to the manufacturer's protocol using the quantiTect Reverse Transcription Kit (Qiagen, The USA). The Real Time PCR was performed by the SYBER Green PCR Master Mix Reagent. The cycling parameters were 95°C for 10 min, then, 40 cycles at 95°C for 15 s, and 60°C for 60 s. The housekeeping β-Actin was normalized.

### 3.4. Toxicology Assay

The viability of treated cells was quantified spectrophotometrically by the 2, 3-bis (2-methoxy-4- Nitro-5-sulfophenyl)-S-| (phenylamino) carbonyl|-2-tetrazoliumhydroxide (XXT) assay (Sigma- Aldrich) (21) . Results were expressed as a percentage of cell viability with respect to control absorbance (100% cell viability). The XTT assay showed that the LA, CLA, EPA and synthetic ligands have no effect on the cell viability in THP-1 in comparison with the control group (data not shown).

### 3.5. Cellular Cholesterol Measurement

Cells were washed twice with Phosphate Buffer Saline (PBS) and homogenized in 200 μl hexane-isopropanol (3:2) (22). The organic phase was used for the TC and EC measurement by enzymatic assays (Calbiochem, The USA). FC was measured as the difference between TC and EC.

The Bradford assay was used for the measurement of cellular proteins (23), and the results were presented as μg lipids/ mg cellular proteins.

### 3.6. Statistical Analysis

All results were presented as Mean ± SD. Quantitative variables in groups were compared by one-way ANOVA, with Tukey multiple comparison test. The homogeneity of variance assumption for ANOVA was checked. The level of significance for all statistical analyses was set at 0.05. Analysis was performed using SPSS software.

## 4. Results

### 4.1. Cellular TC, EC, FC and %EC in Macrophages

**Figure 1.**
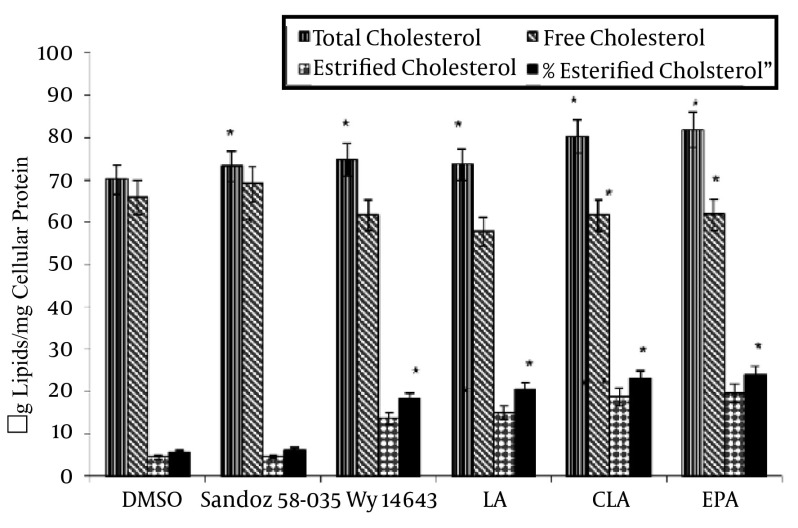
Effects of Fatty Acids on The Intracellular Total Cholesterol (TC), Free Cholesterol (FC) and Esterified Cholesterol (EC) Levels in THP-1 Derived Macrophages and Foam Cells.

Incubation of fatty acid and synthetic ligands could not prevent increasing in the cellular TC. FC was increased by Sandoz58-035 (P = 0.024), and decreased by fatty acid and Wy14643 (P = 0.035). Sandoz58-035 could inhibit increasing in the EC concentration (P = 0.038), in comparison with the Sandoz58-035, and control group. The concentration of EC in the fatty acids groups increased significantly (P = 0.028). The distribution pattern of %EC is similar to the EC pattern. This means that Sandoz58-035 could prevent the increased cellular EC and %EC but in other groups EC and %EC significantly increased (Figure 1). 

THP-1 derived macrophages were cholesterol-loaded with Ac LDL (50 μg/ mL) for 48 hours. Wy14643 (50 μmol/L) and Sandoz58-035 (5 μM) were added 24 hours before cholesterol- loading. TC and FC were enzymatically determined and CE was calculated as the difference between TC and FC. Results are the Mean (SD) of triplicate determinations, representative of 3 independent experiments. Statistically significant differences between treatments were indicated by one-way ANOVA followed by the Tukey multicomparison test. Compared to the controls, all treatments were significant at 0.05 level control Wy14643, Sandoz 58-el.

### 4.2. Molecular Markers of Macrophage Cholesterol Metabolism

**Figure 2.**
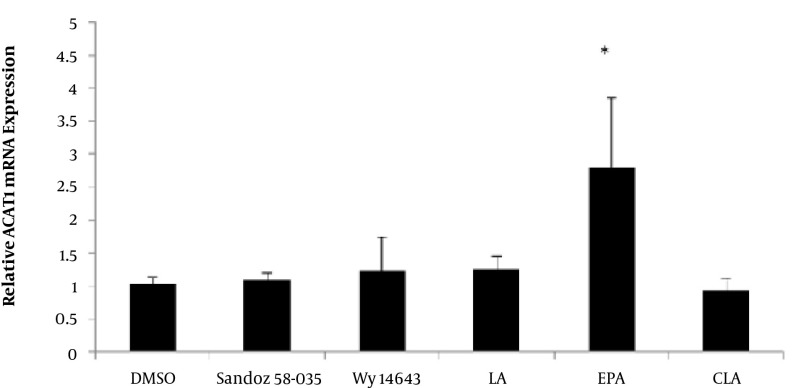
The Effects Of Fatty Acids (100 µM), Wy 14643 (50 µmol/L) and Sandoz58-035(5 µM) on mRNA Levels of ACATI in THP-1 Macrophages Derived Foam Cells.

**Figure 3.**
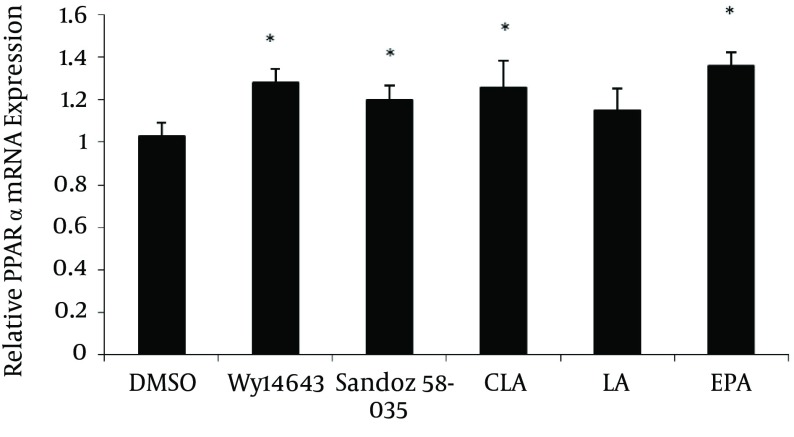
The Effects Of Fatty Acids (100 µM) and Pharmacological PPARα and ACATI Ligands on mRNA Levels of PPARα in THP-1 MACROPHAGEs Derived Foam Cells. *P ≤ 0.05.

Figures 2 and 3 show the effects of LA, CLA, and EPA on the PPARα and ACAT1 mRNA expression, in comparison to WY-14643 (a PPARα agonist) and Sandoz 58-035 an ACAT1 inhibitor). The ACAT1 mRNA expression was significantly increased by EPA (P = 0.009). However, c9, t11- CLA, LA, Wy14643 and Sandoz 58-035 had no significant effect on the mRNA level of ACAT1 expression compared to DMSO (Figure 2). 

Cells were treated for 48 h with fatty acids and pharmacological compounds, and the mRNA levels were analyzed by cyber green procedures. The values were normalized to β-actin. All results represent means of ± SD from triplicate determinations, representative of 3 independent experiments compared to control. Significant differences between treatments were indicated by one-way ANOVA followed by the Tukey multicomparison test. In comparison to the control Wy14643, Sandoz 58-035, c9, t11-CLA, and EPA increased PPARα mRNA levels (P = 0.024, P = 0.041, P = 0.043, P = 0.004 respectively); although, LA had no significant effect on the PPARα mRNA expression (P = 0.489) (Figure 3). 

Cells were treated for 48 h with fatty acids and pharmacological compounds. The mRNA levels were analyzed by cyber green procedures and the values were normalized to β-actin. All results represent means of ± SD from triplicate determinations, representative of 3 independent experiments compared to the control. Significant differences between treatments were indicated by one-way ANOVA followed by the Tukey multicomparison test. 

### 4.3. Molecular Markers of Macrophages and Foam Cells Cholesterol Metabolism

The ACAT1 mRNA expression was significantly increased by EPA (P = 0.009) but c9, t11- CLA, LA, Wy14643 and Sandoz 58-035 had no significant effect on the mRNA level of ACAT1 expression compared to the control. Compared to control, Wy14643, Sandoz 58-035, c9, t11-CLA and EPA, increase PPARα mRNA levels (P = 0.024, P = 0.041, P = 0.043, P = 0.004 respectively); although, LA had no significant effect on the PPARα mRNA expression (P = 0.489) (Figure 3). Also data showed that these fatty acids increase the mRNA level of PPARα when compared to Sandoz58-035. 

## 5. Discussion

Foam cell derived macrophages are key players involved in the initiation and progression of atherosclerosis (24). THP-1 monocyte-macrophages were previously established as a valuable model for studying the lipid homeostasis in human macrophages (25, 26) and have been used in this study to evaluate the effects of natural and pharmacological ligands in lipid metabolism of MQs and MQ-derived foam cells. These cells were formed when MQs uptake many modified LDL particles via the scavenger cells (25, 26). Many studies have shown that n-3 (27-29) and Conjugated fatty acids (CLA) have roles in prevention of progression and promoting process of atherosclerosis, and thus are useful as potent antiatherogenic nutrients in vivo (30). Although other researchers, like Mondy, et al. showed the role of the proatherogenic potential of CLA in C57BL/6 mice (31). In our previous study, we showed that these fatty acids can reduce the cholesterol content of macrophage derived foam cells (14). In the present study, we investigated the effects of the n-3fatty acids and CLA on the prevention of foam cells formation (cholesterol distribution and some molecular factors) in THP-1 derived macrophage cells. In this study we showed that the incubation of cells with fatty acids, and synthetic ligands could not prevent elevation in the intracellular TC. FC was increased by Sandoz58-035 (an ACAT inhibitor) and decreased by a fatty acid, and Wy14643. Sandoz58-035 inhibited the elevation in intracellular EC concentration. In comparison to Sandoz 50-035, the concentration of EC in fatty acids groups increased significantly. The pattern of distribution of %EC was similar to the EC pattern. This means that Sandoz58-035 (ACAT1 inhibitor) could prevent the increased intracellular EC and %EC but in others groups, intracellular EC and %EC increased. These data were similar to the results obtained by Weldon et al. (19). However McLaren JE et al., showed that EPA and DHA reduce ac-LDL uptake (EPA increased ox-LDL uptake) (32), and this effect is due to the activation of PPARα, and consequently its target genes specially scavenger receptor CD36, and this results in the increased uptake of oxLDL. Chinetti et al. showed that preincubation of MQs with Wy14643 did not modify macrophage AcLDL-induced total cholesterol accumulation. However, they found that cholesterol distribution changes with a significant decrease of the CE fraction (20). The increase in CE is due to an increased expression of the ACAT1 gene, because the ACAT1 mRNA levels increased by fatty acids. However Sandoz 58-035 and Wy14643 did not alter the gene expression of ACAT1, but these compounds have effects on the gene expression or activity of other enzymes such as fatty acid synthase, carnitine palmitoyl transferase 1(CPT-1), CD36, scavenger receptor-A, stearoyl CoA desaturase 1 (33), and scavenger receptor-independent mechanism (32).

These researches indicated that fatty acids such as EPA and DHA are capable of regulating the macrophage foam cell formation (33). Also some studies have suggested that the cell-specific manner gene expression of ACAT1 and effect of fatty acids on the ACAT1 mRNA stability may lead to various levels of ACAT1 mRNA expression in different studies. In the present study, the ACAT1 mRNA expression was significantly increased by EPA, but c9, t11 CLA, LA, Wy14643, and Sandoz 58-035 had no significant effect. PPARα is another important gene in lipid metabolism in foam cells which we studied in our research (32, 34). PPARα, γ, and δ are expressed in a variety of metabolic tissues and cell types, including epithelial, Endothelial and immune cells, reflecting their pleiotropic functions in fatty acid homeostasis (15). PPARα is an important lipid-activated transcription factor whose transcriptional target genes have many roles in lipid and glucose metabolism and inflammatory processes (34). Many studies have shown that dietary fatty acids did not significantly change the mRNA level of PPARα in THP-1 derived macrophages, foam cells, and other tissues (19, 35-37). But other studies have indicated opposite results (38, 39). Some of studies have shown that different fatty acids could have different effects on the gene expression of PPARα (14). In macrophages, the transcriptional target of PPARα (such as CPT-1) regulates the balance between free cholesterol and cholesterol esters (19). So the activation of PPARα by natural or synthetic ligands can induce the expression of enzymes which reduces the intracellular TC and EC (14, 20, 40).

Our findings showed that Wy14643, Sandoz 58-035, c9, t11-CLA, and EPA increased the PPARα mRNA levels, but LA had no significant effect on the PPAR α mRNA expression. These effects are different with those observed in our previous study (14). But it is important to understand that the stimulation of cholesterol efflux to the plasma membrane by other factors such as genes regulated by LXR (41), sterol regulatory element binding protein 1 (SREBP1) (42) may have an important role in a reduced intracellular cholesterol as a substrate for ACAT1, thus a reduction in the intracellular TC and EC content (43). Macrophages and macrophages derived foam cells are both crucial targets for the regression of atherosclerosis. The results of this study indicate the ability of these fatty acids in the alteration of cholesterol distribution in macrophage and foam cell formation. Importantly, the results presented provide significant evidence that macrophages and macrophage derived foam cells are critical cellular targets of CLA, n-3 fatty acids, and thus provide new avenues of investigation which may elucidate their exact mechanism in atherosclerosis process. Also we considered that different fatty acids, such as ligands, have different affinity for transcription factors or enzymes, thus they have different effects on the lipid metabolism in foam cells. At this point, we can explain the different results obtained by various researchers. Also one ligand (for example n-fatty acids) affects the cell metabolism by several mechanisms which can be very complicated (42), and this can be explained by different results obtained by various researchers.
